# Some Historical and Chemical Facts Relative to the Welding of Gold

**Published:** 1903-09-15

**Authors:** Henry W. Morgan


### SOME HISTORICAL AND CHEMICAL FACTS RELATIVE TO THE WELDING OF GOLD.

BY HENRY W. MORGAN, M.D., D.D.S.

The following quotations from the printed proceedings of the last meeting of the Mississippi Dental Association, held at Biloxi, June 20–22 (the first from a paper on metallurgy, and the last two from the discussion of it), are my apology for selecting the subject of this paper.

The first, to be found on page 20, is as follows: "I have spent much time in experimenting with different kinds of gold now on the market, and find that soft gold is better than cohesive, *because of its purity.*"

The next statement I desire to combat is found on page 29: "Non-cohesive gold foil is gold in its purest form. The property of cohesion is due to the presence of some foreign substance, though one may not be able to detect it." And the last is contained on page 30, and is in reply to the statement made by one of the members to the effect that Dr. W. H. Morgan years ago proved to his own satisfaction that the fumes of ammonia would have this effect (prevent cohesion), and is clothed in the following language: "I am perfectly familiar with Dr. Morgan's views on the subject, but think that it was pure supposition on his part, and that he did not claim to '*know* anything about it.' "

How such reckless statements went unchallenged I shall not, of course, attempt to explain. That such errors should have gone without some attempt to correct them is to be greatly wondered at. When W. H. Morgan made a statement of fact he had the clear knowledge of what he was doing, and the proof back of it, if necessary. To show how far wrong these gentlemen are is the task I have imposed upon myself this evening.

I do not offer any of this paper as original matter, but confess to have used not only ideas but words, and in some instances whole sentences and even paragraphs, from Dr. G. V. Black's published and unpublished writings, and those of other writers, some of which I have attempted to condense and paraphrase.

Until the year 1855 "cohesiveness" was not recognized as a property of gold foil. There were no allusions to it or literature upon the subject until accidentally discovered by Dr. Robert Arthur, who immediately saw the value of the property and began to talk and write about it.

Two years after, in 1857, he published a book on the subject entitled "Adhesive Gold Foil." I regret that I have not had access to it, that I might have learned from it more of what he knew upon the subject. His discovery has worked a wonderful revolution in operative dental surgery, in extending its benefits and possibilities in the direction of substitution of lost tissues and the restoration of tooth form. The exacting nature of this form of gold has done much to develop the skill of the dental profession to its present high standard of excellence.

It has been declared by a writer of note to be "the stimulative astringent of the dental profession, keeping our operations keyed up to the highest point of efficiency by reason of its imperious demand upon our ability."

The profession has been trained to do other kinds of delicate work as the result of skill acquired in the manipulation of gold, "which training has been the saving grace of dentistry."

The demand of the dental profession for pure gold foil was but the recognition of the earlier operators of the superior working properties of such; then came the demand that the gold beaters should furnish foil very clean; and in the process of arriving at cleanliness of gold and purity combined, several manufacturers about the same time discovered that their gold freshly made and annealed was sticky. This stickiness was considered a disadvantage, and gave the gold manufacturers a great deal of trouble. It was a book of this sticky gold that Dr. Arthur got hold of, and he wrote asking if he could get more. The assertion that fillings could be made with gold which would weld when cold created a considerable stir in the profession, and it was regarded as some peculiar process of manufacture.

From the early sixties until the first part of the seventies this welding property of gold was the subject of much discussion, many articles being written upon it, trying to account for it upon various theores.

In 1869, Dr. G. V. Black read a paper on the subject before the Illinois State Dental Society, setting forth his experiments and conclusions, as to the wonderful property of gold, which paper was published in the *Missouri Dental Journal* and in the *American Dental Review* and in dental journals in Germany and France. The subject was rewritten for the New York Odontological Society; republished by it in 1874, and by the *Dental Cosmos*, Vol. XVII., p. 139, March, 1875. Since then we have heard but little of it. All works treating it, however, make reference to these articles, and no writer has yet dared to take an opposing view. I shall endeavor to give in Dr. Black's language the conclusions arrived at, condensing as much as I can.

"Take a piece of fairly fresh charcoal, and it will absorb into its pores many volumes of carbonic acid gas. This is called capillary attraction. The gas is condensed in the capillaries of the charcoal. These capillaries constitute a vast extent of the surface of the coal, and, in fact, the carbonic acid gas is attracted to the surface and is condensed upon it." This is an example of what I should rather call "surface attraction" than "capillary attraction."

Another somewhat familiar experiment in the chemical laboratory is to take spongy platinum, make a little ball of it on a wire, anneal this thoroughly, let it get cold, and turn a stream of hydrogen gas upon it. In a few minutes the platinum begins to glow, becomes white hot, and the gas will ignite. This spongy platinum, and indeed all platinum, has the power of condensing large quantities of hydrogen gas, and in the condensation of oxygen and hydrogen gas upon the platinum, the liberation of the latent heat of these gases heats the platinum to a white heat and ignites the gas. If the pores of the spongy platinum are filled with an inert substance, the platinum does not heat, but water is formed when the gas is turned on it.

Gold is peculiar among metals in this—that it does not attract oxygen to its surface. It does attract hydrogen feebly. It attracts ammonia strongly. If gold attracts to its surface a gas, and that gas is condensed upon the surface, forming a film, that film will prevent the two surfaces from coming in contact. Hence it will fail to weld. If we succeed in driving that film from the gold by annealing, we will regain the welding property.

These general facts with regard to gold may readily be illustrated by a few simple experiments which any one can perform. Ammonia is strongly attracted to gold. Dr. Black's description of a simple test is as follows: "Place a small quantity of spirits of ammonia, or of aqua ammonia, in a large glass jar. The ammoniacal gas from this will fill the space above the liquid. Now take a rope of gold which has been annealed, and the welding property of which is perfect, and swing it with a thread above the liquid in the jar and replace the cork. In fifteen minutes remove the gold and try its welding property. It will not weld any more than so much tissue paper. If it is swung above chlorine water, the welding property will be completely destroyed in two minutes. Now reanneal these ropes of gold, and the welding property is completely restored."

How are we to know that this effect is produced by a sensation of gas on the surface of the gold? Place the gold first in chlorine gas for ten minutes, and then transfer it to ammonia for an equal time. Now, as these two gases unite to form a volatile salt, ammonium chloride, which readily crystallizes upon any cold substance, place the gold thus treated in a long test tube and heat it quickly over a Bunsen burner. Immediately white fumes begin to leave the gold, and then crystallize in a white ring on the colder portion of the test tube. Chemical examination of these crystals shows them to be ammonium chloride. This could occur only by the condensation of the gases on the gold, and the amount formed shows this condensation to be in very considerable quantity. The experiment may be varied by placing the gold first in the gaseous ammonia, and then transferring it to the chlorine, but in this case there will not be so large an amount of the ammonium chloride formed, for the reason that the ammonia is not condensed on the gold in so large a quantity as the chlorine.

In this experiment the salt formed is volatile, and the gold is readily cleaned by heat. But if the salt formed is a fixed salt that we can not volatilize by the annealing temperature, the welding property of the gold is permanently destroyed. This is what often occurs when the gold is not well protected. Now, one of the principal reasons why the crystalline forms of gold are more difficult to keep in good condition than foil is the fact that the crystals form a sponge that more readily takes up and holds gases.

I will endeavor to show this by repeating Dr. Black's experiment.

"I wish to announce this general principle or fact," says Dr. Black. "All metals weld when brought into contact. All metals are sticky when brought into contact. If they fail to stick when brought into apparent contact, it is because there is something between them that prevents actual contact. We cannot weld platinum, for the reason that it attracts oxygen energetically to its surface. Therefore, in our atmosphere the freshly cut surface is quickly covered with a film of oxygen, which prevents its welding. We cannot weld lead unless we get it together very quickly after it is cut. Cut a piece with a knife, and watch the surface. We can see the film spreading over it. If we can anticipate that by putting them together quickly with sufficient pressure to bring them into contact, we can weld pieces of lead. Also, in drilling into a piece of lead you will notice that the chips stick together, ball up, and give trouble. More or less of this happens when you drill gold or even platinum. This is simply because in the quick working of the drill you bring the pieces into contact while they are yet clean. I have succeeded also in demonstrating this in the machine shop with both steel and iron."

We find that chlorine gas is attracted very strongly to gold, but only when the surface is dry. Sulphuric acid is attracted quite strongly to gold, in fact, all the sulphur group, also the phosphorus group; and if they meet with any substance, whether in gaseous form or not, while residing on the surface of the gold, form a non-evaporable salt which permanently destroys the welding property of gold.

Ammonia forms salts that are distilled away by annealing. If a gas has taken possession of the suface of that gold, and meets with another and forms a salt that is not evaporable by the heat of annealing, the welding property of the gold is permanently lost.

Carbon dioxide is attracted feebly, but after remaining a considerable time on the surface of the gold it is difficult to remove by annealing, and it is probable that there are some combinations of the carbon group that become immovable by annealing, because we find that our gold, when subjected to carbon dioxide, then left to the air for a time, becomes permanently non-cohesive. Nothing less, then, than remelting and refining can give us again the welding property.

The secret of welding cold gold lies in the fact that it does not attract or condense oxygen. Nitrogen (the other element of our atmosphere, which some poetical chemist has characterized as "that slothful element that never knew a passion, neither of hate nor yet of love") is, I believe, not attracted by any of the metals. Therefore, gold does not lose its welding property in pure air, or, if so, it is only after many days, and then, we believe, it is always from the condensation of carbonic acid gas, which is always present in small quantities, and is attracted to gold quite feebly, but with sufficient power to remove the welding property promptly if placed in pure gas. Therefore, gold left exposed to the ordinary atmosphere loses its property in a few days, but it may be retained for a great while by very close wrapping. The effect of annealing, so far as the development of the welding property is concerned, is simply the cleansing of its surface by driving off any volatile gases that may be condensed upon it. This is a very different effect from that of softening gold plate by annealing. The softened plate is not again hardened by exposure to the air or any of its impurities, while the welding property is so lost. The welding property is not so necessarily lost by hammering or even burnishing, while its softness is inevitably lost.

Therefore these two effects of heat are entirely different in their nature. The one cleans the surface, while the other probably causes a repolarization of the atoms. I should probably state that by hammering gold the power of the weld is weakened in proportion to the weakening of the general cohesion of the atoms of the mass. It is well known to workers in gold that it may be hammered till it becomes frail and brittle, and its particles, having lost their cohesion. or attraction for each other, will, of course, have lost in the same degree their attraction for fresh masses, and the weld will be weakened to this extent. It is therefore injurious to continue malleting our fillings after all spaces are closed.

I have already occupied more of your time than was my intention. I started out to correct some statements and to excite an interest on the subject here; to urge the members of this club to make these experiments for themselves, and to demonstrate that it is within their power to make at will from the same sheet of gold foil a filling of "cohesive gold," or a filling of "non-cohesive gold" as well, and that the "purity" of the gold has nothing whatever to do with it. The gold should be pure—yes, as *pure* as it is *possible* to make it—but its *fineness* has nothing to do whatever with its being *cohesive* or *non-cohesive.* The writer holds that a piece of freshly annealed cohesive gold foil is "the softest" form of gold known.

I submit that my father was perfectly familiar with these writings on the subject, and had performed the experiments to demonstrate the results over and over again. Some of you may remember his doing so at Old Point Comfort in 1887, at the meeting of the Southern, with Dr. Stubblefield's assistance.

To permit the statement that it was "pure supposition on his part" to pass without raising my voice to correct it would brand me as unworthy of the name I am so proud to bear.—*Headlight.*

